# Morphology, Molecular Characterization, and Phylogeny of *Travassosius rufus* Khalil, 1922 (Strongylidea: Trichostrongylidae), a Parasite from Endangered Sino-Mongolian Beaver (*Castor fiber birulai*) in Xinjiang, China

**DOI:** 10.3390/ani15091339

**Published:** 2025-05-06

**Authors:** Huiping Jia, Wenwen Chu, Dong Zhang, Kai Li, Wenpu Huang, Xiaoyun Li

**Affiliations:** 1 School of Ecology and Nature Conservation, Beijing Forestry University, Beijing 100083, China; jiahuiping@bjfu.edu.cn (H.J.); 18600646609@163.com (W.C.); 2 Altay Wildlife Conservation Association, Altay 836599, China; hisolak@foxmail.com (W.H.); 18609060935@163.com (X.L.)

**Keywords:** *Castor fiber birulai*, *Travassosius rufus*, phylogeny, mitochondrial genome

## Abstract

The genus *Travassosius* Khalil, 1922, the smallest genus in the subfamily Trichostrongylinae (family Trichostrongylidae), primarily infects the only two extant beaver species worldwide and can be lethal in severe infections. However, the mitochondrial genome evolution of *Travassosius* remains poorly understood, and its phylogenetic placement within Trichostrongylinae is still unresolved. In this study, we applied both morphological techniques (differential interference contrast microscopy) and molecular tools (nuclear ITS2 and mitochondrial genome) to examine *T. rufus* Khalil, 1922. Specimens were collected from the Sino-Mongolian beaver, a subspecies of the Eurasian beaver native to the Ulungur River Basin in northern Xinjiang, China. This work presents the first complete mitochondrial genome sequence and annotation of *T. rufus*, and it is also the first mitochondrial genome reported for the genus *Travassosius*. The mitochondrial genome of *T. rufus* measures 13,646 bp and contains 36 genes, including 12 protein-coding genes (PCGs) (excluding *atp*8), 22 transfer RNA genes, and 2 ribosomal RNA genes. Phylogenetic analysis based on amino acid sequences of 12 mitochondrial PCGs strongly supports the distinctiveness of the genus *Travassosius*. Additionally, *T. rufus* appears to be closely related to *Nematodirus* within Trichostrongylinae. This study also addresses the possible consequences of parasitic infection for the Sino-Mongolian beaver and offers a scientific foundation for conserving this endangered subspecies and managing parasitic diseases in its population.

## Simple Summary

The endangered Sino-Mongolian beaver (*Castor fiber birulai*), a key species for sustaining river ecosystems in China and Mongolia, is threatened by parasitic nematodes. This study focuses on *Travassosius rufus*, a gastric parasite in these beavers, aiming to understand its characteristics and evolutionary background. For the first time, we sequenced the parasite’s mitochondrial genome and compared samples from China with those from Norway and the Czech Republic. Our findings revealed significant genetic divergence among populations, likely due to prolonged geographic isolation. We also found that while *T. rufus* shares ancestry with other parasitic worms, it shows features that are uniquely adapted to its beaver hosts. These results contribute to our understanding of how parasites and hosts evolve together.

## 1. Introduction

Beavers (*Castor* spp.) belong to the order Rodentia and the family Castoridae. Globally, the genus *Castor* includes only two living species: the North American beaver (*C. canadensis*), which is widespread across North America, and the Eurasian beaver (*C. fiber*) [1,2], which once inhabited most river systems throughout northern Eurasia [3]. The Eurasian beaver serves a key ecological function in freshwater systems across Europe and Asia. Its dam-building behavior not only controls water flow [4] but also forms wetland habitats [5], which support greater biodiversity [6,7,8]. The Sino-Mongolian beaver, a subspecies of the Eurasian beaver, is native to the Ulungur River Basin in northern Xinjiang, China, and the adjoining upstream regions along the Mongolian border. It is listed as a first-class nationally protected wild animal in both countries. As a keystone species in the oasis-valley mosaic ecosystem characteristic of arid regions, the Sino-Mongolian beaver plays an essential role in maintaining ecological stability in the region [1,9].

Historically, overhunting of Eurasian beavers for fur and castoreum led to a dramatic population collapse [10]. While conservation efforts have supported the species’ recovery in some areas, health-related risks continue to constrain stable population growth. Among these threats, parasitic infections have emerged as a major concern for Eurasian beaver conservation [11,12]. Parasitic nematodes, which commonly reside in the gastrointestinal tract of mammals, are prominent pathogens. By absorbing nutrients, they may induce malnutrition, anemia, and sometimes severe systemic conditions [13,14].

*Travassosius rufus* Khalil, 1922, a nematode species parasitizing the stomach of the Eurasian beaver, belongs to the family Trichostrongylidae and is the type species of the genus *Travassosius*. In China, this species was first reported by Zhang et al. in 1992 [15]. Subsequent studies have focused mainly on morphological identification. Prior work has shown that nuclear and mitochondrial sequence data, including complete mitochondrial genomes, are essential in the integrative taxonomy, population genetics, and phylogenetic research of Trichostrongylidae nematodes. In recent years, developments in molecular biology have introduced new methods for nematode taxonomy. At present, the NCBI database includes only two mitochondrial COI gene sequences and two nuclear ITS sequences for *T. rufus*, and there are no records of mitochondrial genomes for the genus *Travassosius*. To define the full mitochondrial genome features of *Travassosius*, we sequenced the ribosomal DNA of *T. rufus* obtained from the Sino-Mongolian beaver, including the mitochondrial COI gene and nuclear ITS2 region, and conducted the first annotation of its mitochondrial genome. To evaluate the taxonomic validity of the genus *Travassosius* and explore its phylogenetic placement within Trichostrongylidae, we performed phylogenetic analyses based on concatenated amino acid sequences of 12 mitochondrial protein-coding genes (PCGs), using both Maximum Likelihood (ML) and Bayesian Inference (BI) approaches. This study offers a scientific foundation for conserving the endangered Sino-Mongolian beaver in China and Mongolia and for addressing parasitic infections within its population.

## 2. Materials and Methods

### 2.1. Sample Collection

In May 2024, a Sino-Mongolian beaver rescued from the wild due to malnutrition and extreme emaciation died at the Fuyun County Terrestrial Wildlife Epidemic Prevention and Rescue Station (46.99419326° N, 89.55346159° E). A necropsy was conducted with the assistance of professional veterinarians, with particular attention given to examining the digestive tract for parasitic infections. Nematode specimens collected from the stomach were rinsed in physiological saline (0.9% NaCl) and preserved in 75% ethanol for morphological and molecular analyses. All recovered nematodes were identified as belonging to a single species. Samples were stored at −40 °C for DNA extraction and subsequent analyses.

### 2.2. Morphological Observation

Specimens were cleared with lactophenol to improve the visibility of internal structures. The prepared samples were mounted on glass slides and covered with coverslips. The morphological traits of adult worms were observed and documented under a Carl Zeiss differential interference contrast microscope (Axioscope 5, Baden-Württemberg, Germany) at 10× and 40× magnifications. The recorded characteristics included body length, body width, esophagus length, and sex-specific traits such as male and female tail morphology. All measurements were expressed in micrometers (μm), and statistical analyses were conducted to assess inter-individual variability. We then examined the adult nematodes under a microscope and recorded the number of nematodes.

### 2.3. Molecular Analysis

#### 2.3.1. DNA Extraction, PCR Amplification, and Sequencing

Adult nematode samples stored at −40 °C were used for total genomic DNA extraction using the DNeasy Blood and Tissue Kit protocol. Specific primers targeting multiple genetic markers (18S, ITS, cox1) were designed (Table 1), and PCR amplification was carried out to obtain the corresponding fragments. PCR products were verified using 2% agarose gel electrophoresis and sent to BGI (Beijing Genomics Institute, Beijing, China) for Sanger sequencing. In addition, whole-genome sequencing was performed on the Illumina NovaSeq 6000 PE150 platform using remaining amplified DNA. Sanger sequencing results were employed as bait sequences, and the resulting data were used for genome annotation and bioinformatic analysis.

#### 2.3.2. DNA Annotation and Data Analysis

##### Sequence Analysis

Gene sequences obtained via Sanger sequencing from BGI were compared to the NCBI database using BLAST (Basic Local Alignment Search Tool, v 2.13.0) to identify homologous sequences. Relevant sequences from closely related species within the same genus were downloaded for downstream analysis. Multiple sequence alignments were conducted using the ClustalW algorithm implemented in MEGA 7.0 under default settings. Manual inspection was carried out to exclude incomplete or low-quality terminal regions, ensuring reliable alignments. To reduce background noise and retain informative segments, alignments were trimmed to preserve conserved regions with high-quality signals. Pairwise genetic distance matrices were generated for the 18S rRNA sequences to assess intraspecific variability.

Following sequence alignment and trimming, phylogenetic analysis was performed using the Maximum Likelihood (ML) method on ITS and COI sequences. The Tamura-Nei (TN93) model was selected as the best-fit substitution model. Node support was evaluated through bootstrap analysis with 1000 replicates. The resulting phylogenetic tree was visualized and edited using ITOL (https://itol.embl.de/, accessed on 26 March 2025).

Sequencing data were subjected to quality control, de novo assembly, and annotation via the IDBA assembler. Bait sequences were applied to extract the mitochondrial genome. Partial mitochondrial genome data from other Trichostrongylidae species were obtained from NCBI for comparative purposes. The annotated mitochondrial genome of *Travassosius rufus* was visualized using the Proksee Server (https://proksee.ca/, accessed on 10 December 2024). Further mitochondrial genome analysis included the assessment of nucleotide composition and codon usage across the 12 protein-coding genes (PCGs), excluding stop codons. Relative synonymous codon usage (RSCU) was calculated following the approach of Tamura et al. (2011) [19] using MEGA. AT and GC skews were computed based on the method by Perna and Kocher (1995) [20]. Graphs and figures were produced using the ggplot2 package (v3.4.1) in R and visualized using Prism GraphPad (Prism 9, Boston, MA, USA).

### 2.4. Phylogenetic Tree Construction

Phylogenetic analysis was carried out for both *Travassosius* species and other Trichostrongylidae nematodes to clarify the evolutionary placement and taxonomic distinctiveness of *T. rufus*. Twelve protein-coding gene (PCG) fragments were selected for this purpose. Species from the Cooperiidae family, including *Cooperia oncophora*, along with members of the Habronematidae family, were used as outgroups (Supplementary Table S1, [21,22,23,24,25,26,27,28,29,30,31,32,33,34,35,36,37,38,39,40,41,42,43]). Maximum Likelihood (ML) analysis was performed using RAxML version 8, while Bayesian Inference (BI) analysis was carried out with MrBayes version 3.2.7a, [44]. The resulting phylogenetic trees were used to investigate systematic relationships within Trichostrongylidae. Individual PCG fragments were aligned with MAFFT (version 7.037), concatenated using SequenceMatrix (version 1.7.8), and visualized on the iTOL online platform (https://itol.embl.de/, accessed on 26 March 2025). In addition to tree construction, host–parasite associations were examined. Host data were compiled, including the relationship between *T. rufus* and Sino-Mongolian beaver observed in this study, along with previously reported host records for Trichostrongylidae species from public databases. The taxonomic positions of parasitic species were defined using both ML and BI trees, and host–parasite interactions were mapped accordingly to illustrate these relationships more clearly.

## 3. Results

### 3.1. Morphological and Taxonomic Study of Travassosius rufus in Sino-Mongolian Beaver Based on Classification Methods

**Figure 1:**
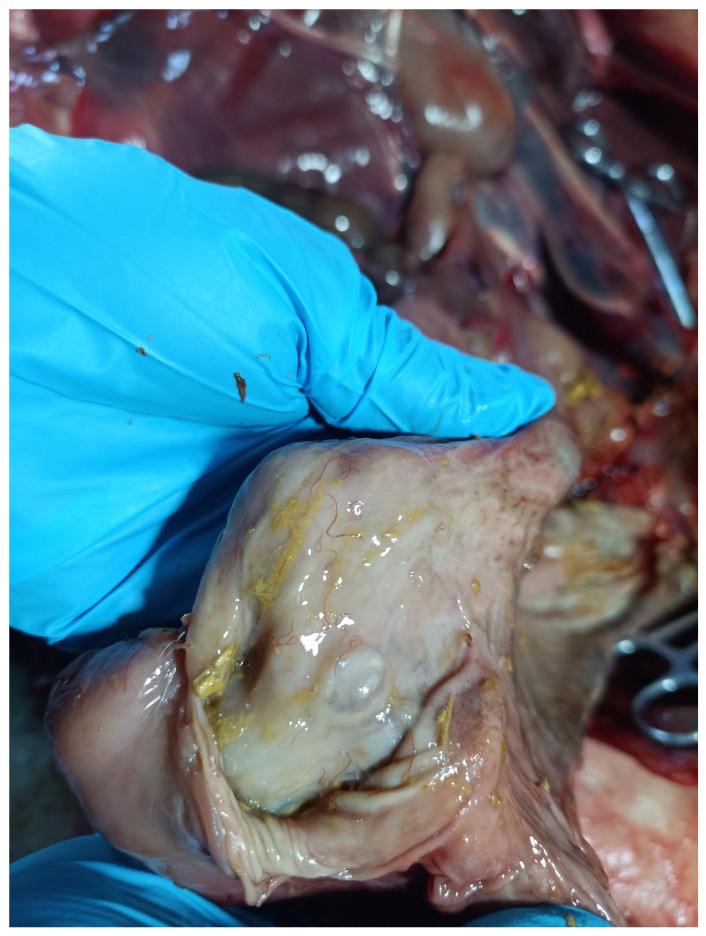
*Travassosius rufus*, a parasitic nematode recovered from the stomach of a deceased Sino-Mongolian beaver individual.

**Figure 2:**
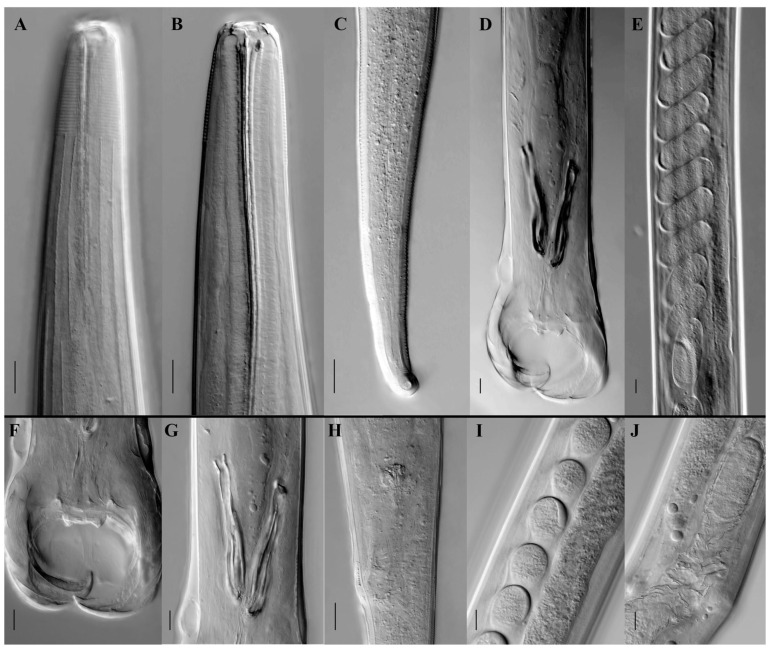
Differential interference contrast micrographs of *Travassosius rufus* (Strongylida: Trichostrongylidae) from the endangered Sino-Mongolian beaver. (**A**,**B**) Head features of *T. rufus*; (**C**) female tail; (**D**) male tail; (**E**) morphology of eggs inside the female; (**F**) male bursal; (**G**) male copulatory spicules; (**H**) female vulva; (**I**) lateral view of eggs inside the female; (**J**) female oviduct and uterus. Scale bars: 20 μm/20 μm/20 μm/50 μm/100 μm/20 μm/20 μm/20 μm/20 μm/20 μm.

A total of 5478 adult nematodes were recovered from the stomach of the Sino-Mongolian beaver during dissection (Figure 1). Morphological identification confirmed the species as *T. rufus*, distinguished by its small, slender form and brick-red coloration. The body is cylindrical, with the greatest width located just behind the midpoint. The anterior and posterior ends of the cuticle show distinct transverse striations, while the rest of the body surface bears longitudinal ridges (Figure 2A). The buccal capsule is small (Figure 2B), and the cervical papillae are prominent. The pre-bursal papillae are large and well-defined, with a broad base and rounded apex. In males, the lateral bursal rays are elongated, while the dorsal rays are comparatively short. A pair of spicules is present, but there is no gubernaculum. Female specimens exhibit a tail that gradually narrows and curves dorsally at the end (Figure 2C). The distance from the tip of the tail to the vulva is shorter than the distance from the vulva to the anus.

Male specimens (based on measurements and observations from three mature individuals; Table 2, Figure 2): Body length ranges from 8.69 to 10.477 mm (mean: 9.68 mm), with a maximum body width of 0.112–0.184 mm (mean: 0.140 mm). The buccal capsule is simple. The cuticle presents 31–32 transverse striations and 30–32 longitudinal ridges. The esophagus measures 0.452–0.552 mm (mean: 0.510 mm), accounting for 4.31–6.35% (mean: 5.27%) of total body length. The nerve ring is situated 0.272–0.340 mm (mean: 0.292 mm) from the anterior end, and the excretory pore lies 0.310–0.385 mm (mean: 0.330 mm) from the anterior end. Cervical papillae are positioned 0.396–0.479 mm (mean: 0.428 mm) from the anterior tip. The bursa margin is undulated, with relatively large lateral lobes and smaller dorsal lobes that are not clearly separated. Lateral lobes typically curve ventrally, forming an embracing pattern. Pre-bursal papillae are large and distinct, measuring approximately 0.015 mm in length, with a broad base and a rounded apex. The paired spicules (Figure 2D,G) are thick and split into two branches near the mid-region. The lateral branch runs closely alongside the median branch, with their distal ends nearly touching. This feature sets *T. rufus* apart from its congener *T. americonus*, where the lateral and median branches of the spicules are more widely spaced. A gubernaculum is absent. The copulatory cone is stout and nearly conical, with numerous bubble-like projections on its surface.

Female specimens (based on measurements and descriptions of three mature individuals; Table 2, Figure 2): Body length ranges from 9.89 to 11.87 mm (mean: 11.24 mm), with maximum body width between 0.142 and 0.161 mm (mean: 0.157 mm). The cuticle shows 31–32 transverse striations and 32–36 longitudinal ridges. The esophagus measures 0.512–0.559 mm (mean: 0.544 mm), accounting for 4.31–5.65% (mean: 4.84%) of total body length. The nerve ring is located 0.260–0.282 mm (mean: 0.271 mm) from the anterior end, and the excretory pore lies 0.332–0.396 mm (mean: 0.358 mm) from the anterior tip. Cervical papillae are situated 0.436–0.468 mm (mean: 0.454 mm) from the anterior end. The vulva is positioned 2.247–2.831 mm (mean: 2.649 mm) from the tail tip (Figure 2H). The tail is long and thin and curves dorsally at the posterior end, terminating in a blunt tip. Transverse striations are visible along the tail surface. Eggs are elongated-oval in shape (Figure 2E,I), with a thin, smooth shell, measuring 78.10–89.56 μm (mean: 81.89 μm) in length and 40.18–52.78 μm (mean: 44.10 μm) in width (Table 2, Figure 2).

### 3.2. Molecular Taxonomy and Phylogenetic Position of Travassosius rufus in Sino-Mongolian Beaver Based on Sanger Sequencing

The genetic variation of nematodes infecting the Sino-Mongolian beaver was analyzed using the 18S rRNA gene. All 18S sequences showed high similarity, with a length of 1577–1578 bp, indicating strong sequence conservation, consistent with the gene’s role as a core ribosomal component. A genetic distance matrix revealed very low divergence among most samples (0.000–0.003), particularly between samples 2, 5, and 6, where the distance was 0.000 (Supplementary Table S2), suggesting a genetically homogeneous or clonal origin. However, sample 1 showed a significantly greater genetic distance (0.016–0.018), exceeding the typical intraspecific variation threshold (>1%). This could suggest potential taxonomic divergence (e.g., a cryptic species) or technical artifacts such as sequence contamination.

**Figure 3:**
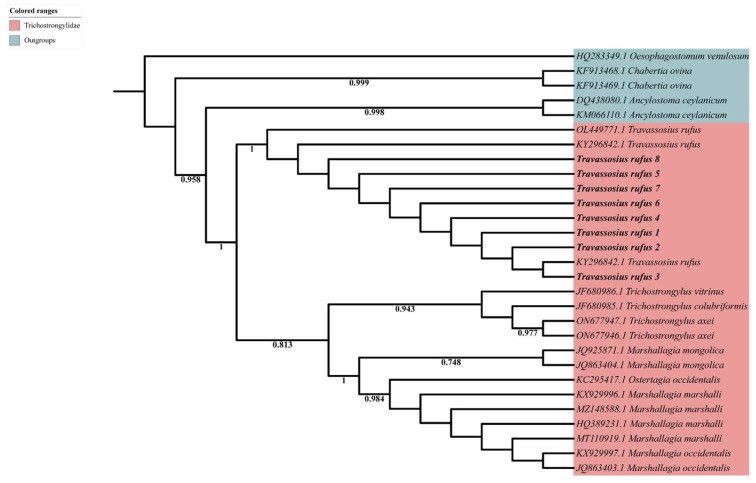
Maximum likelihood (ML) inference based on the ITS2 sequence data showing the phylogenetic relationships of representatives of Trichostrongylidae. *Ancylostoma ceylanicum* (Rhabditida: Ancylostomatidae), *Oesophagostomum venulosum* (Rhabditida: Strongylidae), and *Chabertia ovina* (Rhabditida: Chabertiidae) were chosen as the outgroups. Bootstrap values ≥ 70 are shown in the phylogenetic trees. Bold indicates *Travassosius rufus* in this study.

Sanger sequencing revealed that the ITS2 gene sequence of *T. rufus* samples was 419–455 bp in length. Alignment with *T. rufus* sequences available in the GenBank database showed consistently high similarity, exceeding 97.97%. Similarity with other sequences was below 90%. A phylogenetic tree based on nuclear ITS2 gene sequences (Figure 3) was constructed to examine the genetic relationships among *T. rufus* samples from various geographic locations. Tree branches were color-coded by family for visualization. *Travassosius rufus* samples (*T. rufus* 1–8) from the Sino-Mongolian beaver in China clustered with previously published sequences from *C. fiber* in Norway (KY296842.1, KY287762.1) and the Czech Republic (OL449771.1), with strong support (bootstrap = 1), indicating high sequence similarity across regions. Phylogenetic analysis placed *T. rufus* in a distinct and well-supported clade within Trichostrongylidae, as sister to *Trichostrongylus* (support = 0.813), and clearly separated from *Marshallagia* and *Ostertagia.* No evident clustering by host or geography was observed among *T. rufus* samples, indicating low intraspecific variation in the ITS2 region and supporting its status as a valid, phylogenetically distinct species.

**Figure 4:**
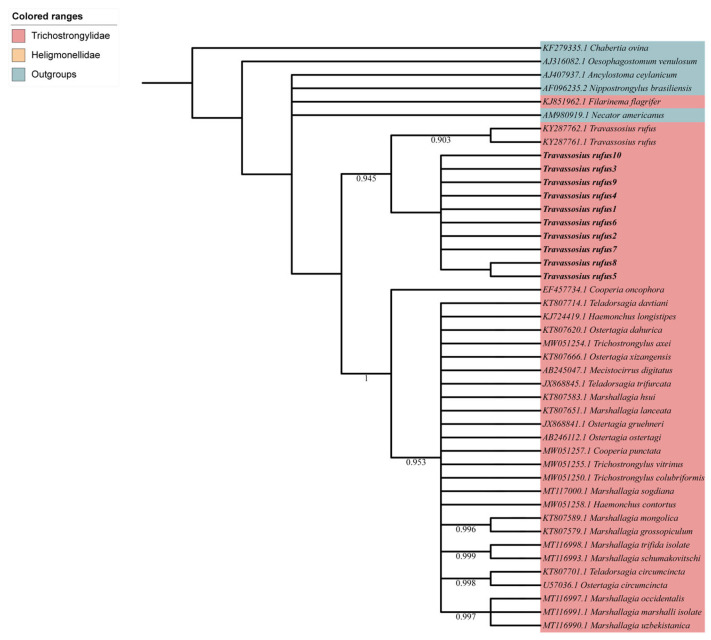
Maximum likelihood phylogenetic tree based on mitochondrial COI sequences. Maximum likelihood (ML) inference based on the COI sequence data showing the phylogenetic relationships of representatives of Trichostrongylidae. *Ancylostoma ceylanicum* and *Necator americanus* (Rhabditida: Ancylostomatidae), *Oesophagostomum venulosum* (Rhabditida: Strongylidae), *Chabertia ovina* (Rhabditida: Chabertiidae), and *Nippostrongylus brasiliensis* (Rhabditida: Heligmonellidae) were chosen as the outgroups. Bootstrap values ≥ 70 are shown in the phylogenetic trees. Bold indicates *Travassosius rufus* in this study.

The phylogenetic tree based on mitochondrial COI sequences (Figure 4) was constructed to explore the evolutionary relationships among *T. rufus* samples and related nematode species. Consistent with the ITS2-based findings, multiple *T. rufus* sequences clustered into a strongly supported monophyletic branch (support value = 0.903), indicating molecular coherence and evolutionary separation at the species level. *T. rufus* samples from the Sino-Mongolian beaver in China grouped into a clade alongside sequences from Norway (KY287761.1, KY287762.1), suggesting substantial genetic similarity among geographically distant populations. This branch has a clear differentiation boundary from other genera and species in the Trichostrongylidae family (indicated by the pink background), such as *Trichostrongylus*, *Ostertagia*, and *Marshallagia*. In the phylogenetic tree, the branch where *Travassosius rufus* did not form a sister group relationship with any specific genus but exists as an independent evolutionary lineage within the Trichostrongylidae family suggests that its phylogenetic position is relatively independent and is further supported at the molecular level based on morphological classification. The outgroups *Ancylostoma ceylanicum*, *Necator americanus*, *Oesophagostomum venulosum*, *Chabertia ovina*, and *Nippostrongylus brasiliensis* successfully anchored the root of the tree, supporting the overall phylogenetic structure and evolutionary relationships among the species analyzed.

### 3.3. Phylogenetic and Micro-Taxonomic Classification of Travassosius rufus Based on Whole-Genome Sequencing

**Figure 5:**
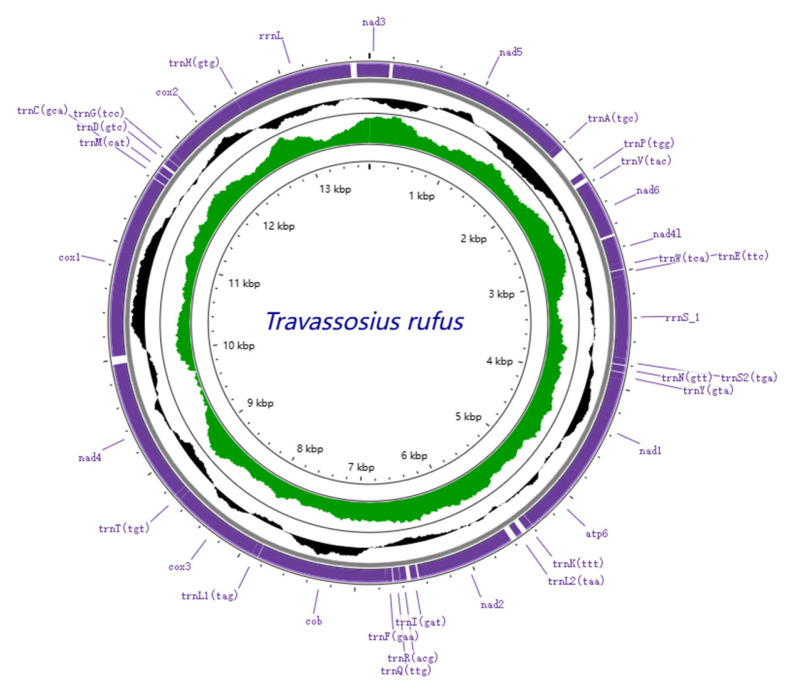
Schematic representation of the mitochondrial genome of *Travassosius rufus* isolated from Sino-Mongolian beaver.

**Figure 6:**
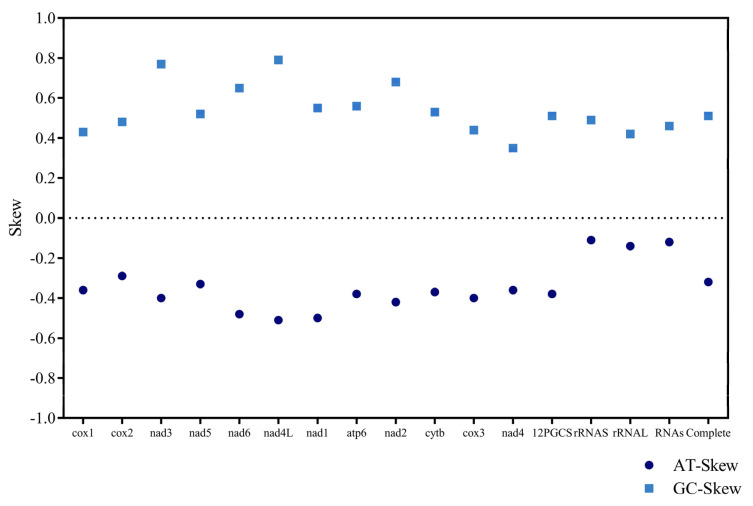
Mitochondrial genome composition and base bias in *Travassosius rufus* from Sino-Mongolian beaver.

**Figure 7:**
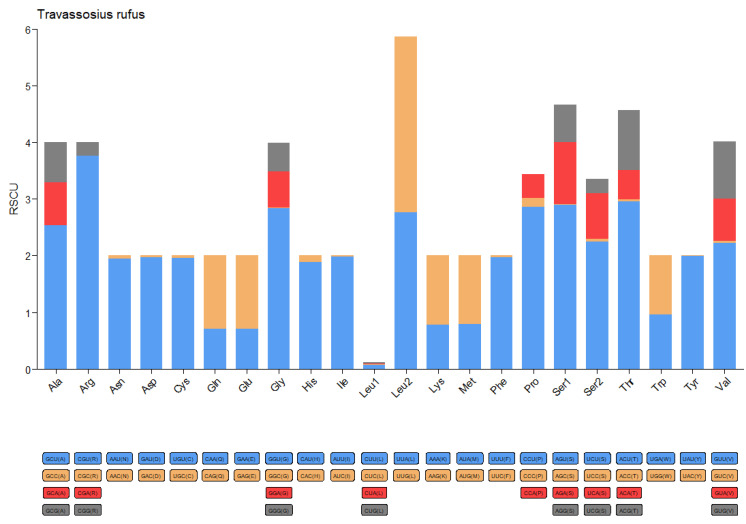
Codon usage patterns in the mitochondrial genome of *Travassosius rufus* found in Sino-Mongolian beaver.

The complete mitochondrial genome of *Travassosius rufus* was sequenced, revealing a total length of 13,755 bp. It includes 12 protein-coding genes (*cox*1–3, *nad*1–6, *nad*4*L*, *atp*6, *atp*8, and *cytb*), 22 tRNA genes, and 2 rRNA genes (Table 3, Figure 5). The mitochondrial genome size of *T. rufus* is comparable to those of other Trichostrongylidae species, such as *Trichostrongylus colubriformis* (13,653 bp) and *Trichostrongylus vitrinus* (13,800 bp). All genes are encoded on the H-strand (positive strand) (Table 4, Figure 5). The mitochondrial DNA of *T. rufus* shows a strong AT bias (74.0%), with thymine (T) being the most frequent nucleotide (51.1%) and cytosine (C) the least (6.0%). The overall AT-skew and CG-skew are −0.314 and 0.510, respectively. The combined length of the 12 protein-coding genes is 10,296 bp, making up 75.9% of the total mitochondrial genome (Table 4). These genes also show a high AT content, ranging from 67.5% to 77.2%, with *nad*6, *nad*4, and *nad*5 showing the highest A+T content at 76.4%, 76.7%, and 77.2%, respectively (Table 4). The AT-skew values for *nad*6, *nad*4, and *nad*5 are −0.48, −0.36, and −0.33, and the GC-skew values are 0.65, 0.35, and 0.52, respectively (Table 4, Figure 6). Together, the 12 protein-coding genes encode 3429 amino acids (excluding stop codons). Codon usage analysis identified CGU (Arg), UUG (Leu), and ACU (Thr) as the most frequently used codons in the *T. rufus* mitochondrial genome (Figure 7). Additionally, 22 typical tRNA genes were annotated, ranging from 55 to 64 bp in length, all encoded on the H-strand. The mitochondrial genome also contains two rRNA genes: rRNAS (703 bp) and *rrn*L (982 bp). The *rrn*S gene is situated between *tRNA-Glu* and *tRNA-Ser2*, while *rrn*L lies between *tRNA-His* and the protein-coding gene *nad*3. The A+T content of *rrn*S and *rrn*L is 74.8% and 77.8%, respectively, with an overall RNA A+T content of 76.3%.

**Figure 8:**
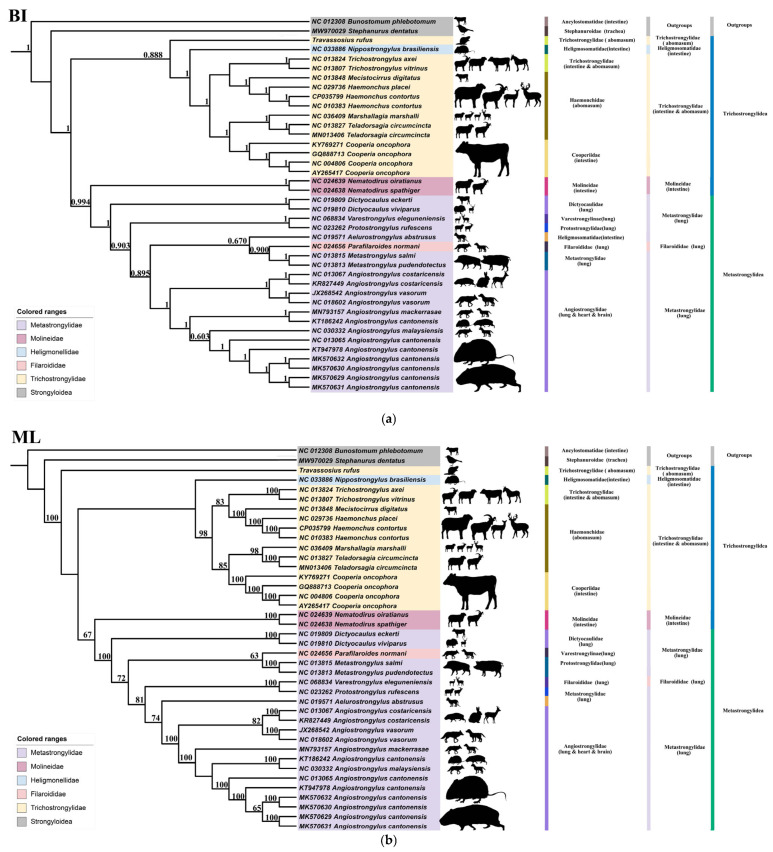
Phylogenetic relationships among Trichostrongylidae and Metastrongylidea nematodes inferred from BI (**a**) and ML (**b**) methods based on the amino acid sequences of 12 PCGs of mitochondrial genomes. *Bunostomum phlebotomum* (Rhabditida: Ancylostomatidae) and *Stephanurus dentatus* (Rhabditida: Stephanuroidae) were chosen as the outgroup. Bootstrap values ≥ 70 and Bayesian posterior probabilities values ≥ 0.70 are shown in the phylogenetic trees. BI, Bayesian inference; ML, maximum likelihood; PCGs, protein-coding genes.

This study constructed BI and ML phylogenetic trees based on 12 mitochondrial PCGs. The tree topologies produced by both methods were highly consistent, clearly resolving phylogenetic relationships across families such as Trichostrongylidae, Metastrongylidae, and Haemonchidae, and forming well-supported monophyletic clades. In the BI tree, *T. rufus* was positioned within Trichostrongylidae and grouped as a sister taxon to *Nipostrongylus brasiliensis*, with a support value of 0.888. This suggests that *T. rufus* may share a recent common ancestor with *N. brasiliensis* or exhibit similar host-related evolutionary traits. The placement of *T. rufus* aligned with other Trichostrongylidae members, reinforcing its classification within this family (Figure 8a). In the ML tree, *T. rufus* also appeared within Trichostrongylidae and again formed a sister group with *N. brasiliensis* (Figure 8b), confirming the consistency of the BI results. These outcomes demonstrate that the assignment of *T. rufus* to Trichostrongylidae is stable and its evolutionary relationships are consistently supported across different analytical methods. Compared to other species within Trichostrongylidae, *T. rufus* occupies a separate evolutionary branch, indicating it may have undergone distinct evolutionary changes linked to host adaptation.

## 4. Discussion

### 4.1. Taxonomy of Travassosius rufus in Beavers Based on Morphology

The genus *Travassosius* comprises two recognized species: *T. rufus* Khalil, 1922, and *T. americanus* Chapin, 1925 [45], which parasitize the stomachs of Eurasian and North American beavers, respectively. However, the classification of these two species has long been debated. Cameron (1938) regarded *T. americanus* as a junior synonym of *T. rufus*, arguing that both names refer to the same species [46]. In contrast, researchers such as Smith (1967) maintained that *T. rufus* and *T. americanus* should be treated as distinct species [47]). In 1978, Bush and Samuel analyzed specimens collected from Alberta, Pennsylvania, British Columbia, and Europe [48]. By comparing the type specimens of *T. americanus* and *T. rufus*, they identified pronounced morphological differences between parasites infecting North American and Eurasian beavers. In particular, they noted distinct differences in the length and structure of the male spicules, supporting the recognition of *T. americanus* and *T. rufus* as separate species. They further proposed that *T. americanus*, which infects North American beavers, represents the ancestral lineage, while *T. rufus*, found in Eurasian beavers, emerged through geographic isolation. Their work also raised the possibility that the *T. americanus* population on Vancouver Island might be undergoing speciation [48]. Based on the morphological traits described by Zhang Meiyun et al. and Bush et al., we identified the nematodes collected from the Sino-Mongolian beaver in this study as *T. rufus* [15,48]. Further confirmation was achieved using differential interference contrast (DIC) microscopy, which allowed for clearer observation of diagnostic features such as transverse rings on the body surface, longitudinal striations, spicules, and egg morphology. This approach enabled a more detailed evaluation of key taxonomic characteristics, reinforcing the identification of *T. rufus*.

### 4.2. Molecular Taxonomy and Phylogenetic Status of Travassosius rufus Based on DNA Barcoding

Based on the research results, this study revealed the genetic characteristics of *T. rufus* and its taxonomic status within the family Trichostrongylidae from a molecular phylogenetic perspective. Phylogenetic analyses based on the nuclear ITS2 and mitochondrial COI sequences both demonstrated that *T. rufus* formed a strongly supported monophyletic clade within Trichostrongylidae, with clear evolutionary boundaries separating it from closely related genera such as *Trichostrongylus*, *Ostertagia*, and *Marshallagia*. These molecular findings corroborate the validity of *T. rufus* as a distinct species, originally established through morphological characteristics. Notably, despite differences in evolutionary rates between ITS2 and COI markers, both genes revealed high intraspecific sequence conservation in *T. rufus*: ITS2 sequences exhibited >97.97% similarity across geographically distributed populations, while COI clustering indicated low genetic divergence among populations from China, Norway, and the Czech Republic. This pattern suggests that *T. rufus* may possess strong host adaptability, with its dispersal potentially linked to host migration history and niche conservatism.

In phylogenetic reconstructions, *T. rufus* did not form a sister-group relationship with any specific genus but instead occupied an independent evolutionary lineage within Trichostrongylidae, possibly reflecting its unique evolutionary trajectory or the existence of undisclosed closely related taxa. The outgroup species (e.g., *Oesophagostomum venulosum* and *Chabertia ovina*) successfully anchored the root of the phylogenetic tree, enhancing the reliability of the topological structure, though caution is warranted regarding the limitations of mitochondrial genes in resolving deep-level phylogenetic relationships. Overall, the genetic data obtained for *T. rufus* in this study will serve as a critical reference for future research on species identification, population genetics, and phylogenetic investigations of this taxon.

### 4.3. Molecular Taxonomy and Phylogenetic Status of Travassosius rufus Based on Genome Sequencing

This study presents the first complete mitochondrial DNA (mtDNA) genome of *T. rufus* parasitizing the Sino-Mongolian beaver in China. Comparative analysis with other Trichostrongylidae species, such as *Trichostrongylus colubriformis* (13,693 bp) and *Trichostrongylus axei* (13,653 bp), showed that the mtDNA genome length of *T. rufus* is similar, indicating a degree of size conservation across the family. Nucleotide composition analysis exhibited a strong AT bias (73.9%) in the *T. rufus* mtDNA genome, especially within PCGs, where the A+T content reached 75.9% (Table 4). Notably, nad5 (77.2% AT) and nad6 (76.4% AT) exhibited the highest AT content, with negative AT-skew (−0.33 and −0.48, respectively) and positive GC-skew (0.52 and 0.65), reflecting strand-specific mutational pressures from unidirectional replication and limited DNA repair [49,50,51]. This compositional bias aligns with observations in other nematodes [52,53] and may facilitate metabolic adaptation to parasitic niches by reducing transcriptional energy costs [54] and tolerating mutation accumulation under host oxidative stress. Similar to other members of Trichostrongyloidea, including *Mecistocirrus digitatus* (15,221 bp) [21] and *Haemonchus contortus* [55], the *T. rufus* mitochondrial genome lacks the *atp*8 gene. All 12 PCGs in *T. rufus* are encoded on the heavy strand and transcribed in the same direction, consistent with findings in other Trichostrongylidae species (see Figure 8). The PCGs of *T. rufus* mainly use standard start codons such as ATT and TTG, with common termination codons including TAA and TAG. Some genes show alternative patterns: *cox*2, *nad*3, *cytb,* and *nad*4 begin with GTT, ATG, ATA, and GTG, respectively. In several cases, genes like *nad*5 end with incomplete stop codons (e.g., T). These incomplete stop codons are typical in nematode mitochondrial genomes [25,26].

In the mitochondrial DNA genome of *Travassosius rufus*, certain codons—such as CGU (Arg), UUG (Leu), and ACU (Thr)—show a clear usage preference. This pattern is consistent with findings from studies on the *Haemonchus* genus and other nematodes [26,28]. The codon usage bias (e.g., CGU/UUG dominance) and high AT content in rRNA genes (76.3% overall) further align with energy-efficient genome streamlining, balancing rapid adaptation and functional conservation [24,56]. This supports the research of Gendron et al. (2024) [57], where niche-specific pressures (e.g., parasitism) and phylogenetic constraints jointly shape nematode mitogenome plasticity [51,54]. Codon preference analysis in this study highlights differences in gene expression control and protein synthesis between *T. rufus* and other nematode species. These distinctions suggest that *T. rufus*, as a strict parasite of the Sino-Mongolian beaver, may have developed unique adaptive traits over its evolutionary history, allowing it to remain well-suited to varying host environments. Both Bayesian Inference (BI) and Maximum Likelihood (ML) analyses in this study place *T. rufus* within the family Trichostrongylidae, forming a sister group with *Nipostrongylus brasiliensis*. This indicates the possibility of a shared common ancestor. This taxonomic position is in line with earlier morphological classifications, further supporting the stable placement of *T. rufus* within Trichostrongylidae. The evolutionary trajectory of *T. rufus* may have been shaped by host specificity or environmental selection. Further investigation is needed to better understand its host adaptation and ecological dynamics.

The host specificity of *T. rufus* and its possible evolutionary route are particularly noteworthy. *T. rufus* primarily infects beavers (Castoridae), which contrasts with the usual host range of most Trichostrongylidae species. For example, *N. brasiliensis* parasitizes rodents, while many other Trichostrongylidae species infect ruminants or equids. In both the BI and ML phylogenetic trees, *T. rufus* appears closely related to *N. brasiliensis*, suggesting that it may represent a specialized lineage adapted to rodent hosts. The adaptation of *T. rufus* to beavers could be the result of a host-switching event, or it is possible that its ancestors infected a broader range of hosts before gradually specializing in beavers. This study presents new molecular evidence for the systematics of *T. rufus*, based on its mitochondrial genome and the full set of 12 protein-coding genes. Mitochondrial genomes, due to their relatively high mutation rates and maternal inheritance, provide strong resolution in phylogenetic studies of parasitic nematodes. The agreement between BI and ML results supports the reliability of mitochondrial data for resolving the phylogenetic position of *T. rufus*. Future work should incorporate nuclear gene markers (e.g., ITS, 18S, and 28S rRNA) to refine the evolutionary framework and clarify the lineage history of *T. rufus*.

## 5. Conclusions

This study investigates the structural and genomic features of *Travassosius rufus*, a parasitic nematode exclusive to the endangered Sino-Mongolian beaver, through morphological, genetic, and phylogenetic analyses. It fills a key gap in mitochondrial genome data for *T. rufus*. Phylogenetic trees constructed using ML and BI approaches confirm that *T. rufus* from the Sino-Mongolian beaver shows significant genetic separation from other species. Its evolutionary history suggests a pattern of co-evolution with its host. DNA markers (e.g., ITS2, *cox*1) identified here offer practical tools for non-invasive monitoring of parasite prevalence and genetic diversity, critical for detecting infection risks during population recovery efforts. Given the strong host specificity of *T. rufus*, uncontrolled parasite loads may exacerbate conservation challenges for this keystone species. Integrating genomic insights into management strategies—such as routine parasite screening in introduction programs—could mitigate declines. Future studies should prioritize quantifying the link between *T. rufus* infection intensity and host health while exploring parasite adaptation mechanisms to refine conservation measures. These findings underscore the importance of parasite surveillance and investigation in wildlife, particularly for ecologically pivotal hosts like the Sino-Mongolian beaver.

## Figures and Tables

**Table 1:** Details of primers used for PCR amplification of target regions in this study.

Gene	Primer Name	Primer Sequence (5′→3′)	References
18S	18S-F	AAAGATTAAGCCATGCA	[16]
	18S-R	GCAGGTTCACCTACAGAT	
ITS	ITS-F	GAGTCGATGAAGAACGCAG	[17]
	ITS-R	GAATCCTGGTTAGTTTCTTTTCCT	
COI	COI-F	TTTTTTGGGCATCCTGAGGTTTAT	[18]
	COI-R	TAAAGAAAGAACATAATGAAAATG	

**Table 2:** Detailed information on morphological characteristics of the *Travassosius rufus*.

Measurement Item	*Travassosius rufus*	
	Female	Male
Number (individuals)	3	3
Body length (mm)	9.89–11.87	8.69–10.477
Body width (mm)	0.142–0.161	0.112–0184
Esophagus length (mm)	0.512–0.559	0.452–0.552
Nerve ring to head (mm)	0.260–0.282	0.272–0.340
Excretory pore to head (mm)	0.332–0.396	0.310–0.385
Cervical papillae to head (mm)	0.436–0.468	0.396–0.479
Vulva to tail tip (mm)	2.247–2.831	——
Size of eggs (μm)	78.10–89.56 × 40.18–52.78	——

**Table 3:** Gene organization of the complete mitochondrial genome of *Travassosius rufus*.

Gene	Start	End	Length	Strand	Gap or Overlap	Start Codon/Stop Codon
*cox*1	1	1578	1578	+	8	ATT/TAG
tRNA-Cys	1585	1640	56	+	10	
tRNA-Met	1649	1706	58	+	23	
tRNA-Asp	1728	1785	58	+	6	
tRNA-Gly	1790	1845	56	+	2	
*cox*2	1846	2541	696	+	1	GTT/TAA
tRNA-His	2541	2596	56	+	3	
*rrn*L	2598	3561	964	+	4	
*nad*3	3564	3899	336	+	30	ATG/TAG
*nad*5	3928	5509	1582	+	2	ATT/T
tRNA-Ala	5510	5565	56	+	255	ATT/TTT
tRNA-Pro	5819	5872	54	+	40	
tRNA-Val	5911	5966	56	+	1	
*nad*6	5966	6400	435	+	20	TTG/TAA
*nad*4L	6419	6649	231	+	3	ATT/TTT
tRNA-Trp	6651	6707	57	+	9	
tRNA-Glu	6715	6770	56	+	−2	
*rrn*S	6767	7471	705	+	2	
tRNA-Ser2	7472	7524	53	+	12	
tRNA-Asn	7535	7590	56	+	8	
tRNA-Tyr	7597	7651	55	+	1	
*nad*1	7651	8526	876	+	−3	TTG/TAA
*atp*6	8522	9121	600	+	5	ATT/TAA
tRNA-Lys	9125	9186	62	+	48	
tRNA-Leu2	9233	9288	56	+	2	
tRNA-Ser	9289	9340	52	+	2	
*nad*2	9341	10,183	843	+	15	TTG/TAA
tRNA-Ile	10,197	10,255	59	+	35	
tRNA-Arg	10,289	10,343	55	+	5	
tRNA-Gln	10,347	10,401	55	+	10	
tRNA-Phe	10,410	10,465	56	+	2	
*cytb*	10,466	11,578	1113	+	5	ATA/TAA
tRNA-Leu1	11,582	11,636	55	+	2	
*cox*3	11,637	12,404	768	+	0	ATT/TGT
tRNA-Thr	12,403	12,457	55	+	2	
*nad*4	12,458	13,687	1230	+		GTG/TAA

**Table 4:** Nucleotide composition and skew values of different regions in the mitochondrial genome of *Travassosius rufus*.

Regions	Size(bp)	T(U)	C	A	G	AT(%)	GC(%)	GT(%)	AT Skew	GC Skew
*cox*1	1576	45.9	9.3	21.6	23.1	67.5	32.3	69	−0.36	0.43
*cox*2	696	45.0	7.8	25.0	22.3	70.0	30.1	67.3	−0.29	0.48
*nad*3	336	51.8	3.0	22.0	23.2	73.8	26.2	75	−0.40	0.77
*nad*5	1561	51.2	5.4	26.0	17.3	77.2	22.7	68.5	−0.33	0.52
*nad*6	435	56.6	4.1	19.8	19.5	76.4	23.6	76.1	−0.48	0.65
*nad*4L	231	57.1	2.6	18.6	21.6	75.7	24.2	78.7	−0.51	0.79
*nad*1	875	51.9	6.9	17.4	23.9	69.3	30.8	75.8	−0.50	0.55
*atp*6	599	50.3	6.0	22.5	21.2	72.8	27.2	71.5	−0.38	0.56
*nad*2	841	53.5	4.0	21.6	20.8	75.1	24.8	74.3	−0.42	0.68
*cytb*	1110	47.8	7.2	21.8	23.2	69.6	30.4	71	−0.37	0.53
*cox*3	768	50.9	7.7	21.6	19.8	72.5	27.5	70.7	−0.40	0.44
*nad*4	1228	52.0	7.6	24.7	15.7	76.7	23.3	67.7	−0.36	0.35
coding 1	3419	48.3	5.1	24.1	22.6	72.3	27.7	70.9	−0.33	0.63
coding 2	3419	47.7	8.7	24.1	19.4	71.9	28.1	67.2	−0.33	0.38
coding 3	3418	55.0	6.1	19.0	19.9	74.0	26.0	74.9	−0.49	0.53
PCGs	10,256	50.3	6.6	22.5	20.6	72.8	27.2	70.9	−0.38	0.51
*rrn*L	967	43.1	5.7	34.5	16.6	77.6	22.3	59.7	−0.11	0.49
*rrn*S	703	42.5	7.3	32.3	17.9	74.8	25.2	60.4	−0.14	0.42
rRNAs	1670	42.9	6.3	33.6	17.2	76.5	23.5	60.1	−0.12	0.46
tRNAs	1232	42.6	5.9	34.8	16.6	77.4	22.5	59.2	−0.10	0.48
Complete mitochondrial genome	13,720	48.6	6.4	25.3	19.7	73.9	26.1	68.3	−0.32	0.51

## Data Availability

The raw sequence for this study has been deposited into the NCBI Sequence Read Archive (SRA) under BioProject PRJNA1199902 with the accession IDs SAMN45892168, Sequence Read Archive: SRR31758216. The complete mitogenome of *Travassosius rufus* is available under the accession number PV530183.

## Supplementary Materials

The following supporting information can be downloaded at: https://www.mdpi.com/article/10.3390/ani15091339/s1, Table S1: Detailed information on representative species from the families Trichostrongyloidea and Metastrongyloidea used in phylogenetic analyses. Table S2: Nucleotide divergence of 18S gene fragment (1577-1578bp) of 12 samples (*Travassosius rufus*).
